# 981. Inducing Antimicrobial Resistance with Long-Term Antibiotics at Stage 2 Revision for Periprosthetic Joint Infection

**DOI:** 10.1093/ofid/ofac492.823

**Published:** 2022-12-15

**Authors:** Judd Payne, Jesse Sutton, Brenna Blackburn, Hannah Imlay, Emily S Spivak, Lucas Anderson, Jeremy Gililland, Jakrapun Pupaibool, Laura Certain

**Affiliations:** University of Utah, Sandy, Utah; George E Whalen Veterans Affairs Medical Center & University of Utah, Salt Lake City, Utah; University of Utah, Sandy, Utah; University of Utah Health, Salt Lake City, UT; University of Utah School of Medicine, Salt Lake City, Utah; University of Utah, Sandy, Utah; University of Utah, Sandy, Utah; University of Utah, Sandy, Utah; University of Utah, Sandy, Utah

## Abstract

**Background:**

Studies have shown reduced rates of recurrence of periprosthetic joint infection (PJI) with the use of oral antibiotics after stage-two revision. We examined the effects of these antibiotics on the antibiotic susceptibility patterns of subsequent PJI.

**Methods:**

We conducted a retrospective cohort study of patients who underwent two-stage exchange for PJI in the Veterans Affairs system from 1/1/2015 – 6/1/2020. We compared patients who were prescribed oral antibiotic prophylaxis for ³14 days following stage-two revision arthroplasty with those who received < 14 days of antibiotics. The primary outcome was the presence of antibiotic-resistant organisms isolated in cultures from subsequent infections of the same joint. Secondary outcomes included comparisons of rates of recurrent PJI and time-to-subsequent PJI between each group. We used Chi square and Fisher exact tests to compare the two groups.

**Figure:**
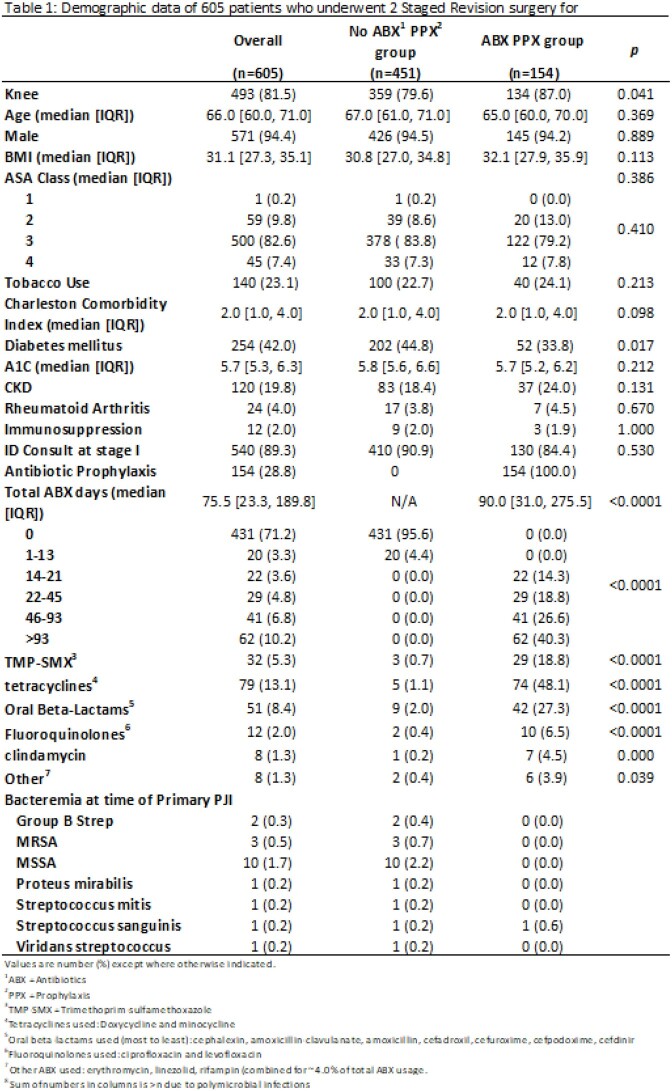

**Results:**

605 patients met inclusion criteria. Of these, 154 received ³14 days of oral antibiotics following stage 2 surgery. Tetracyclines (48.1%), beta-lactams (27.3%) and trimethoprim-sulfamethoxazole, TMP-SMX (18.8%) were the most common antibiotics used. There was decreased susceptibility to tetracyclines (58 vs 73%, p 0.24), TMP-SMX (48 vs 70%, p 0.059), and clindamycin (38 vs 57%, p 0.22) in organisms isolated from subsequent PJI in the antibiotic prophylaxis group compared to the control group. Subsequent PJI was diagnosed in 29 out of 154 (18.8%) in those who received antibiotic prophylaxis, compared with 80 of 451 (17.7%) who did not. Days to subsequent infection was higher for those who received antibiotics compared to those who did not (101 vs 65 days, respectively).

**Figure:**
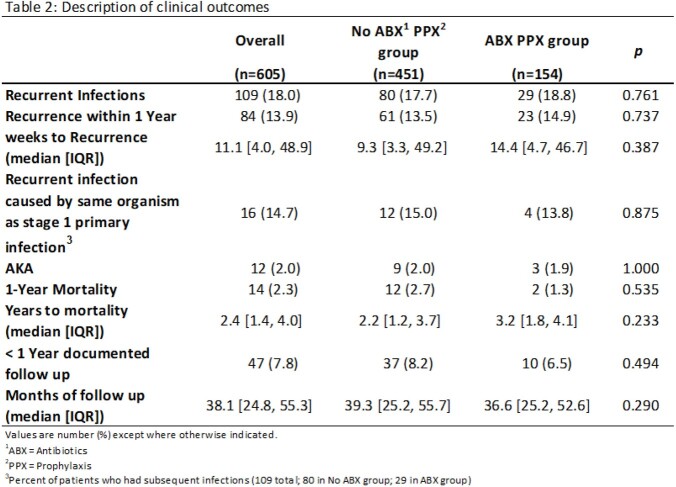

**Figure:**
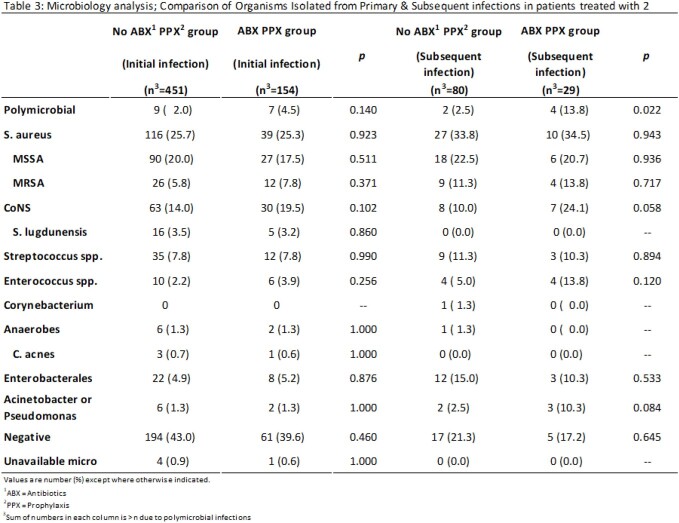

**Figure:**
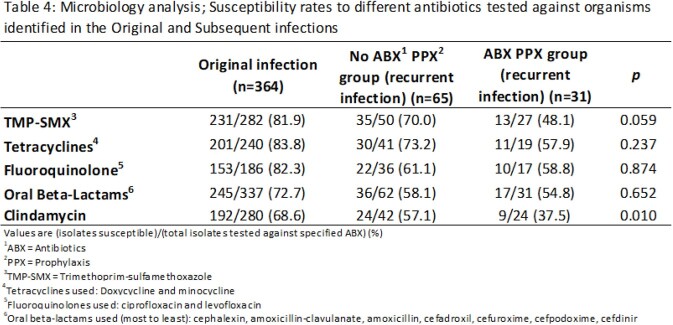

**Conclusion:**

Prophylactic antibiotics following stage 2 revision for PJI may increase the risk for drug-resistant organisms in subsequent index joint infections, most notably against tetracyclines and TMP-SMX. Based on our data, antibiotics given after stage 2 revision postponed, rather than prevented, subsequent infection.

**Disclosures:**

**All Authors**: No reported disclosures.

